# Resilience and coping behaviour among adolescents in a high-income city-state during the COVID-19 pandemic

**DOI:** 10.1038/s41598-023-31147-0

**Published:** 2023-03-11

**Authors:** Aminath Shiwaza Moosa, Ding Xuan Ng, Wai Keong Aau, Wei Teck Timothy Goy, Chenghan Roy Yang, En Hsien Andrew Sim, Juan Dee Wee, Ngiap Chuan Tan

**Affiliations:** 1 SingHealth Polyclinics, Singapore, Singapore; 2 Hwa Chong Institution, Singapore, Singapore

**Keywords:** Human behaviour, Paediatric research, Paediatric research

## Abstract

The study aimed to determine the resilience of multi-ethnic, multi-cultural adolescent students in cosmopolitan Singapore, their coping abilities, and the impact on their social and physical activities during the COVID-19 pandemic and its association with their resilience. A total of 582 adolescents in post-secondary education institutes completed an online survey from June to November 2021. The survey assessed their sociodemographic status, resilience level using the Brief Resilience Scale (BRS) and Hardy-Gill Resilience Scale (HGRS), the impact of the COVID-19 pandemic on their daily activities, life settings, social life, social interactions, and coping ability in these aspects of life. Poor ability to cope with school life (adjusted beta = − 0.163, 95% CI − 1.928 to 0.639, p < 0.001), staying home (adjusted beta = − 0.108, 95% CI = − 1.611 to − 0.126, p = 0.022), sports (adjusted beta = − 0.116, 95% CI − 1.691 to − 0.197, p = 0.013) and friends (adjusted beta = − 0.143, 95% CI − 1.904 to − 0.363, p = 0.004) were associated with statistically significant low resilience level measured with HGRS. About half and a third of the participants reported normal and low resilience, respectively, based on BRS (59.6%/32.7%) and HGRS (49.0%/29.0%) scores. Adolescents of Chinese ethnicity and low socioeconomic status had comparatively lower resilience scores. Approximately half of the adolescents in this study had normal resilience despite the COVID-19 pandemic. Adolescents with lower resilience tended to have lower coping abilities. The study did not compare changes in the social life and coping behaviour of the adolescents due to COVID-19, as data on these aspects prior to the pandemic was unavailable.

## Introduction

The SARS-CoV-2 or Coronavirus Disease 2019 (COVID-19) has resulted in a pandemic that has already claimed more than 6 million lives, with over 645 million confirmed cases of infection from December 2019 to December 2022^1^. The rapid spread of this potentially fatal virus and the emergence of its Variants of Concern have resulted in fear and anxiety, which is detrimental to the mental health and psychological well-being of populations across the world^2,3^. In addition, the various measures to curb and mitigate the spread of the pandemic in the community, such as quarantines and lockdowns, aggravate mental health risks of people across the ages^3^.

Adolescents are susceptible to such risks due to the physical, mental and social changes during this period of their growth and additional pressure from peers and schools in their education environment^4^. They are reported to experience increased depressive symptoms, negative affect, loneliness, and lower academic adjustment, especially during this pandemic^5^. A recent systematic review reveals that younger age and being a student are risk factors for mental health disorders during the COVID-19 pandemic^6^. However, the review did not identify any modifiable factors and coping mechanisms, which can be potentially strengthened to mitigate their mental health risks.

In Singapore, adolescents are typical multi-ethnic Asian students in schools, junior colleges, polytechnics, and junior undergraduates in universities. These adolescents face high stress in the Singaporean competitive educational system^7^. A study by the Organisation for Economic Cooperation and Development (OECD), which conducts the PISA (Programme for International Student Assessment) test, revealed students in Singapore reported higher levels of anxiety about schoolwork compared to those from other nations^8^. COVID-19 pandemic elevated the stress level of adolescents, resulting in profound impact on their mental health, particularly during the circuit breaker period^9^. Most workplaces were closed during this period, and full home-based learning was implemented in primary and secondary schools. This meant working parents of young children had to juggle working from home and performing childrearing duties with little support. Closure of university and college campuses led to local students from dysfunctional homes returning to a potential pressure-cooker environment^10^. These disruptions in academic and social life, uncertainties about examinations and graduation, as well as diminishing career prospects with a looming recession contributed to a sense of fear, worry and anxiety experienced by the students^10^. Recognising the profound psychological impact, the Singapore government has set up a national hotline in April 2020, along with other community-based hotlines to provide emotional and psychological support to the public^11^. Officers managing helplines and other support platforms reported a surge by these adolescents seeking assistance during the pandemic^12^. Such support portals deliver advice and suggestions to enable these adolescents to better cope with their distress and boost their resilience amidst their adverse situations. However, the magnitude and specific areas of their distress, resilience levels and coping mechanisms remain unclear.

Coping comprises cognitive and behavioural strategies to handle and manage stressful events or negative psychological and physical outcomes^13^. Resilience refers to the adaptive capacity to recover from stressful situations in the face of adversity^14^. An Australian study reported that resilience among adolescents during the COVID-19 pandemic were associated with decreased psychological distress and increased positive experiences^15^. Australian adolescents attributed their top three coping strategies to be socialising (38%), hobbies (24%) and physical activities (12%)^15^. A study among Chinese undergraduate students showed that higher psychological resilience was associated with better positive coping behaviour^16^.

These young students affected by this prolonged COVID-19 pandemic will soon transit into adulthood. Evidence has shown that mental health and resilience during adolescence are associated with psychological well-being during adulthood^17^. Literature is limited on the resilience and coping abilities of the western educated, multi-ethnic and multi-cultural Asian students in cosmopolitan Singapore. Understanding their resilience status, coping strategies, and associated risk factors are essential to identify a subset of their peers at higher risk for mental health disorders, especially during a crisis such as the current pandemic. The lessons gained from this crisis will help develop adolescent-centric interventions and inform in contingency planning for the next pandemic.

The findings from this study will raise the awareness of healthcare providers, schools, and policymakers on the psychological health of at-risk adolescents and design programs to enhance their resilience and coping skills. This study aims to assess the resilience level among adolescents in institutes of higher education in Singapore. The objectives of this study are: (1) determine the resilience level among adolescent students in post-secondary educational institutes in Singapore (2) to describe the impact of the COVID-19 pandemic on social and physical activities experienced by these adolescents and their ability to cope in these settings, and; (3) to assess the extent that resilience was associated with this impact and coping ability.

## Methods

### Study design

A cross-sectional questionnaire survey study was conducted during the COVID-19 pandemic to determine the resilience level and coping behavior of adolescents who are students in post-secondary educations institutes in Singapore.

### Participants and setting

Participants were recruited from any post-secondary educational institute in Singapore. These educational institutes included a total of 11 junior colleges (equivalent to high school), 5 polytechnics and 3 institutes of technical education. The study inclusion criteria were adolescents aged 16–19 years, currently enrolled in any post-secondary educational institute and able to complete the online survey in English.

### Recruitment and data collection

Over a 6-month period from June to November 2021, participants were recruited using a web link and a QR code which the investigators shared on social media such as WhatsApp and Instagram. Convenience sampling via networking and snowballing was employed to recruit participants. An anonymous self-administered online survey was used to collect data from the participants. The survey questionnaire was posted on a secure online platform, FormSG, which is available to the public sector and public healthcare clusters in Singapore^18^. Evidence suggests research participants disclose sensitive and personal information, such as mental health symptoms and health behaviour, more frequently when responding to self-administered anonymous questionnaires than when taking part in face-to-face or telephone interviews^19–21^.

### Study instrument

Data on participants sociodemographic, resilience level and impact of COVID-19 on their activities and their coping behavior were collected using online self-administered questionnaire in English language.

#### Sociodemographic data collection form

Participants' year of birth, gender, ethnicity, nationality, housing type (as surrogate indicators of socioeconomic status) and educational institute were collected via the anonymized questionnaire.

#### Resilience measuring questionnaires

Participants' resilience was measured using the Brief Resilience Scale (BRS) and Hardy-Gill Resilience Scale (HGRS).

Brief Resilience Scale*:* BRS is a validated questionnaire that has been used in the local context to assess resilience in mental health professionals^22,23^. It is a six-item instrument that evaluates the ability to recover from stress in any general event. Three items were positively focused, and the remaining were negatively focused. Responses ranged from a score of 1 (Strongly Disagree) to 5 (Strongly Agree) for positively focused questions. The scoring was reverse coded for negatively focused questions. A minimum and maximum score of 6 and 30 could be attained from the six questions, respectively. A BRS score was subsequently obtained from the mean score of the six-item. The BRS Score can be categorized as follows: above 4.3 (High resilience), 3.0 to 4.3 (Normal resilience) and below 3 (Low resilience)^24^.

Hardy-Gill Resilience Scale: HRS has been validated with high test reliability. The questionnaire measures an individual's resilience by understanding their feelings in response to an event or how they felt using a 4-point scale, with nine questions in total. For this study, 'the event' refers to the COVID-19 pandemic between February 2020 and April 2021. The sum of the composite score would generate a score between 0 (least resilient) to 18 (most resilient)^25^.

#### Impact of COVID-19 and coping ability questionnaire

A bespoke questionnaire developed by the authors for the current study was used to assess the impact of COVID-19 on participants' daily activities, life settings, social life, and social interactions.

To measure the impact of COVID-19 on participants' daily activities, participants were presented with seven dichotomous questions (Yes, No), which asked them the activities they conducted during the pandemic and whether they had any past COVD-19 infection. Examples of questions include "Do you study/Work from home?", "Do you order food delivery?", "Do you travel by public transport?".

Participants were asked 25 questions on how their life, social life and social interactions have been affected due to the COVID-19 pandemic and 25 questions on their coping ability in a similar context to the former. These questions on impact include "How severely are you affected? School", "How severely are you affected? Meals (Buying, accessing food)", "How severely are you affected? Family members in the same household", "How severely are you affected? At hawker center". A total of 5 options were available, not applicable, not affected, little affected, affected and badly affected. Some of the questions to assess the participant's ability to cope included "How well are you coping? School", "How well are you coping? Transport", "How well are you coping? Family members staying elsewhere", "How well are you coping? At the wet market". A total of 5 options were available for selection, not applicable, very poorly, poorly, well, and very well.

### Study variables

This study collects sociodemographic and resilience related variables from participants. The sociodemographic variables include age, gender, ethnicity, nationality, and educational institution. They will be used as predictors for resilience score for participants. Similarly, questions that asked participants to rate how much they were affected and how well they were coping in each life situation are used as predictors. This will allow identification of potential gaps in resilience management among students.

### Statistical analysis

Descriptive statistics were used to describe the sample, and correlations were performed to consider relationships between age, gender, ethnicity, institution, and housing type with resilience. The continuous variables were expressed as mean (standard deviation), the categorical as size (percentage). BRS scores were further grouped into low resilience and non-low resilience. The comparison of demographics parameters between low resilience and non-low resilience for association was tested using the Chi-Squared test.

The normality of the continuous data was based on the z-score of skewness and kurtosis, Kolmogorov–Smirnov and Q–Q plot. Based on results from the normality test, investigators used the independent t-test or Mann Whitney U test and ANOVA or Kruskal–Wallis H test, where appropriate. Investigators employed multiple linear regression to determine the predictors of both the Brief Resilience and Hardy-Gill Resilience Score. Variables with p < 0.25 from the univariate analysis were included in the multiple linear regression model to account for potential confounders. The significance of the p-value was set at p < 0.05. Multiple linear regression results were reported as beta coefficient at 95% confidence interval, and p-value. Statistical analysis was performed using SPSS Statistics Version 27.0.

### Ethical consideration

Ethics approval was obtained from the Institutional Review Board in Hwa Chong Institution (ethics approval number is not available). All methods were carried out in accordance with relevant guidelines and regulations. No consent was obtained from the participants in this e-survey to maintain their anonymity, reduce any potential stigma and to gather their truthful responses. The purpose and confidentiality of the study were explained to all participants through the online portal, and their participation was voluntary.

## Results

A total of 582 participants aged 17–20 years participated in the study, with a mean age of 17.3 years (SD = 0.48). A majority were males (64.9%), Chinese ethnicity (80.2%), Singaporean nationality (95.2%), lived in private residential estates (47.7%) and were from junior colleges (75.1%). Participants who indicated Hostel as a residency type (n = 6) were excluded from the subsequent analysis due to the small sample size and difficulty in ordinal categorization for socioeconomic status.

### Sociodemographic variables and resilience score of the participants

BRS mean score is significantly associated with HDB 1–3 Room Flat housing type (adjusted beta = − 0.107, 95% CI − 0.591 to − 0.039, p = 0.025), HDB 4–5 Room Flat housing type (adjusted beta = − 0.124, 95% CI − 0.388 to − 0.067, p = 0.006) and Chinese ethnicity (adjusted beta = − 0.100, 95% CI − 0.432 to − 0.018, p = 0.033). Other sociodemographic variables including gender, nationality educational institutes were not associated with the BRS mean score (Table 1).

**Table 1:** Association between sociodemographics and resilience score.

Variable	Mean (SD) or n (%)	Brief Resilience Scale Score	Unadjusted Beta (95% CI)	Adjusted Beta (95% CI)	P-value	Hardy-Gill Resilience Score	Unadjusted Beta (95% CI)	Adjusted Beta (95% CI)	p-value
		Mean ± SD				Mean ± SD			
Age	17.26 (0.47)	–	–	–	–	–	–	–	–
Gender									
Male	376 (65.3%)	3.22 (0.93)	− 0.03 (− 0.19–0.12)	–	–	9.03 (3.84)	− 0.56 (− 1.24–0.12)	0.001 (− 0.504 to 0.514)	0.985
Female	200 (34.7%)	3.25 (0.85)	Ref	–	–	9.59 (4.12)	Ref	Ref	–
Nationality									
Singaporean	551 (95.7%)	3.22 (0.90)	− 0.08 (− 0.44–0.28)	–	–	9.28 (3.93)	1.24 (− 0.34–2.83)	0.037 (− 0.451 to 1.896)	0.227
Permanent resident	25 (4.3%)	3.30 (0.85)	Ref	–	–	8.04 (4.25)	Ref	Ref	–
Ethnicity									
Chinese	461 (80.0%)	3.21 (0.90)	− 0.08 (− 0.27–0.1) 0.08 (− 0.27–0.1)	− 0.100 (− 0.432 to − 0.018)	0.033*	9.78 (3.90)	2.82 (2.04–3.59)	− 0.012 (− 0.840 to 0.611)	0.757
Non-Chinese^1^	115 (20.0%)	3.30 (0.90)	Ref	Ref	–	6.97 (3.27)	Ref	Ref	–
Housing type									
Private Residential	278 (48.3%)	3.30 (0.87)	Ref	Ref	–	9.16 (4.13)	Ref	–	–
HDB2 4–5 Room^2^	237 (41.1%)	3.20 (0.88)	− 0.1 (− 0.26–0.06)	− 0.124 (− 0.388 to − 0.067)	0.006*	9.38 (3.92)	0.22 (− 0.47–0.9)	–	–
HDB2 1–3 Room^2^	61 (10.6%)	3.00 (1.04)	− 0.3 (− 0.55 to − 0.05)	− 0.107 (− 0.591 to − 0.039)	0.025*	8.86 (3.12)	− 0.3 (− 1.4–0.8)	–	–
Higher Learning Institutes									
Junior College	431 (74.8%)	3.26 (0.89)	Ref	–	–	9.83 (3.97)	Ref	Ref	–
Polytechnic	98 (17.0%)	3.11 (0.94)	− 0.14 (− 0.34–0.06)	–	–	7.75 (3.38)	− 2.08 (− 2.92 to − 1.25)	− 0.075 (− 1.487 to − 0.088)	0.027*
Institute of Technical Education	47 (8.2%)	3.20 (0.91)	− 0.06 (− 0.33–0.22)	–	–	6.70 (2.93)	− 3.13 (− 4.28 to − 1.99)	− 0.027 (− 1.359 to 0.579)	0.429

*p < 0.05.

^1^Non Chinese = Malay, Indian and other ethnicity.

^2^HDB = Singapore Housing Development Board (Public housing type is a surrogate indicator of socioeconomic status, with the more affluent living in larger apartments). Model for BRS is adjusted for ethnicity and housing type. Model for HGRS is adjusted for gender, nationality, ethnicity and education institution.

Average HGRS among participants from junior college were significantly higher than participants from the institute of technical education (9.83 versus 6.70, adjusted beta = − 0.075, 95% CI − 1.487 to 0.088, p = 0.027). Average HGRS for Chinese (9.78) was found to be higher as compared to non-Chinese (6.97). Sociodemographic factors of gender, nationality, ethnicity, and housing type were not associated with the HGRS mean score (Table 1).

### Impact of COVID-19 and ability to cope on daily activities, life settings, social interactions, and social life (Table 2)

**Table 2:** Extent of impact and ability to cope in various life settings.

	Impact					Ability to Cope				
	Badly Affected	Affected	Little Affected	Not Affected	NA	Very Poorly	Poorly	Well	Very Well	NA
COVID-19 on life										
School	37 (6.4%)	342 (59.4%)	177 (30.7%)	20 (3.5%)	–	20 (3.5%)	273 (47.4%)	261 (45.3%)	22 (3.8%)	–
Home	30 (5.2%)	258 (44.8%)	224 (38.9%)	64 (11.1%)	–	21 (3.6%)	218 (37.8%)	266 (46.2%)	71 (12.3%)	–
Sports	37 (6.4%)	225 (39.1%)	248 (43.1%)	66 (11.5%)	–	23 (4%)	190 (33%)	289 (50.2%)	74 (12.8%)	–
Meals	13 (2.3%)	198 (34.4%)	283 (49.1%)	82 (14.2%)	–	13 (2.3%)	157 (27.3%)	317 (55%)	89 (15.5%)	–
Hobbies	17 (3%)	193 (33.5%)	271 (47%)	95 (16.5%)	–	12 (2.1%)	167 (29%)	300 (52.1%)	97 (16.8%)	–
Mask wear	30 (5.2%)	178 (30.9%)	275 (47.7%)	93 (16.1%)	–	17 (3%)	139 (24.1%)	297 (51.6%)	123 (21.4%)	–
Transport	11 (1.9%)	161 (28%)	283 (49.1%)	121 (21%)	–	8 (1.4%)	143 (24.8%)	291 (50.5%)	134 (23.3%)	–
Social interaction										
Boss	69 (12%)	19 (3.3%)	37 (6.4%)	9 (1.6%)	442 (76.7%)	69 (12%)	19 (3.3%)	32 (5.6%)	11 (1.9%)	445 (77.3%)
Colleagues	66 (11.5%)	30 (5.2%)	36 (6.3%)	10 (1.7%)	434 (75.3%)	67 (11.6%)	19 (3.3%)	42 (7.3%)	12 (2.1%)	436 (75.7%)
Community	102 (17.7%)	65 (11.3%)	181 (31.4%)	35 (6.1%)	193 (33.5%)	100 (17.4%)	52 (9%)	198 (34.4%)	33 (5.7%)	193 (33.5%)
Family members (same household)	104 (18.1%)	137 (23.8%)	234 (40.6%)	96 (16.7%)	5 (0.9%)	98 (17%)	96 (16.7%)	284 (49.3%)	90 (15.6%)	8 (1.4%)
Family members (different household)	121 (21%)	140 (24.3%)	193 (33.5%)	56 (9.7%)	66 (11.5%)	98 (17%)	107 (18.6%)	248 (43.1%)	50 (8.7%)	73 (12.7%)
Friends	133 (23.1%)	213 (37%)	187 (32.5%)	35 (6.1%)	8 (1.4%)	111 (19.3%)	147 (25.5%)	244 (42.4%)	65 (11.3%)	9 (1.6%)
Social Life										
School	140 (24.3%)	207 (35.9%)	200 (34.7%)	22 (3.8%)	7 (1.2%)	115 (20%)	155 (26.9%)	265 (46%)	34 (5.9%)	7 (1.2%)
Family life	111 (19.3%)	97 (16.8%)	245 (42.5%)	98 (17%)	25 (4.3%)	108 (18.8%)	64 (11.1%)	266 (46.2%)	112 (19.4%)	26 (4.5%)
Hawker	114 (19.8%)	80 (13.9%)	240 (41.7%)	51 (8.9%)	91 (15.8%)	108 (18.8%)	60 (10.4%)	256 (44.4%)	61 (10.6%)	91 (15.8%)

Ninety-six percent (96%) of participants informed that they attended school-from-home during the pandemic. Food delivery services and public transport were used by 85% and 90% of the participants, respectively. 25% of participants did not engage in any physical activity in indoor and outdoor settings. When broken down into ethnicity, a higher proportion of Chinese (28%) did not exercise than non-Chinese (9%) (Supplementary Fig. S1).

The extend of impact on various life settings and ability to cope in these setting are provided in Table 2. The top three affected aspects of life were school (65%), home (50%) and sports (45%). On the other hand, transportation (30%) was the least affected. COVID-19 had impacted their social interactions with friends (60%) family members from both the same (42%) and different (45%) households. Responses for both colleagues and bosses were the least affected, with the majority (76%) stating it was not applicable. Participants responded that school (62%), family life (36%) and hawker center (34%) were the most impactful aspect that affects their social life. Only 20% of the participants were affected by the cost of mask and availability of mask.

The majority of participants reported coping difficulties in school (51%), home (41%) and sports (37%). They also indicated poor coping with friends (44%) family members from both the same (35%) and different (33%) households. In social life, participants were coping poorly with school (47%), family life (30%) and at the hawker center (29%).

### Brief Resilience Scale Score and its association with impact of COVID-19 and coping ability

Participants had an average score of 3.23 on the BRS. In BRS, the majority of the participants had a normal resilience (59.6%), followed by low resilience (32.7%) and high resilience (7.7%) (Table 3).

**Table 3:** Resilience level of participants.

Brief Resilience Scale (score)*	n (%)	Hardy-Gill Resilience (score)*	n (%)
High (> 4.3)	44 (7.6%)	High (> 14)	125 (21.7%)
Normal (3.0–4.3)	344 (59.7%)	Mid (7–12)	284 (49.3%)
Low (< 3)	188 (32.7%)	Low (< 7)	167 (29.0%)

*Detailed items of the scales with the scoring are provided in the supplementary information.

While 37% of the participants agreed with the question "I have a hard time making it through stressful events", most of the participants (76%) were able to bounce back quickly after setback (Supplementary information Fig. S2).

Impact on life, social interactions or social life were not associated with resilience level. The ability to cope with school life (adjusted beta − 0.123, 95% CI − 0.416 to − 0.029, p < 0.025) and social life in school (adjusted beta − 0.139, 95% CI − 0.484 to − 0.020, p < 0.033) were associated with a statistically significant low resilience level (Tables 4, 5, 6).

**Table 4:** Association between life setting and resilience score.

Variable	Brief Resilience Scale Score	Unadjusted Beta (95% CI)	Adjusted Beta (95% CI)	p-value	Hardy-Gill Resilience Score	Unadjusted Beta (95% CI)	Adjusted Beta (95% CI)	p-value
	Mean ± SD				Mean ± SD			
Impact (school life)								
Affected minimally	3.48 (0.69)	Ref	Ref	–	11.15 (3.14)	Ref	Ref	–
Affected	3.12 (0.96)	0.36 (0.2–0.52)	− 0.057 (− 0.301 to 0.079)	0.250	8.37 (3.97)	2.78 (2.12–3.44)	− 0.029 (− 0.892 to 0.404)	0.460
Ability to cope (school life)								
Well	3.44 (0.69)	Ref	Ref	–	11.18 (3.20)	Ref	Ref	–
Poor	3.02 (1.02)	− 0.42 (− 0.56 to − 0.27)	− 0.123 (− 0.416 to − 0.029)	0.025*	7.33 (3.66)	− 3.85 (− 4.42 to − 3.29)	− 0.163 (− 1.928 to − 0.639)	< 0.001*
Impact (stay home)								
Affected minimally	3.31 (0.76)	Ref	–	–	10.80 (3.11)	Ref	Ref	–
Affected	3.18 (0.97)	0.13 (− 0.02–0.28)	–	–	8.22 (4.09)	2.58 (1.95–3.21)	− 0.057 (− 1.098 to 0.178)	0.157
Ability to cope (stay home)								
Well	3.34 (0.78)	Ref	Ref	–	11.02 (3.43)	Ref	Ref	–
Poor	3.07 (1.02)	− 0.26 (− 0.41 to − 0.12)	− 0.039 (− 0.272 to 0.130)	0.489	6.69 (3.16)	− 4.33 (− 4.88 to − 3.78)	− 0.108 (− 1.611 to − 0.126)	0.022*
Impact (sports)								
Affected minimally	3.28 (0.82)	Ref	–	–	9.97 (3.49)	Ref	Ref	–
Affected	3.19 (0.95)	0.09 (− 0.06–0.24)	–	–	8.66 (4.17)	1.31 (0.66–1.95)	0.020 (− 0.491 to 0.815)	0.627
Ability to cope (sports)								
Well	3.31 (0.82)	Ref	Ref		10.55 (3.63)	Ref	Ref	–
Poor	3.09 (1.01)	− 0.22 (− 0.37 to − 0.07)	− 0.047 (− 0.284 to 0.107)	0.374	6.97 (3.41)	− 3.58 (− 4.18 to − 2.98)	− 0.116 (− 1.691 to − 0.197)	0.013*

*p < 0.05. Model for BRS is adjusted for ethnicity and housing type. Model for HGRS is adjusted for gender, nationality, ethnicity and education institution.

**Table 5:** Association between social interaction and resilience score.

Variable	Brief Resilience Scale Score	Unadjusted Beta (95% CI)	Adjusted Beta (95% CI)	p-value	Hardy-Gill Resilience Score	Unadjusted Beta (95% CI)	Adjusted Beta (95% CI)	p-value
	Mean ± SD				Mean ± SD			
Impact (family-same household)								
NA	3.40 (0.89)	0.11 (− 0.69–0.92)	–	–	6.60 (1.51)	− 3.59 (− 7.03 to − 0.16)	− 0.109 (− 9.323 to 0.019)	0.051
Affected minimally	3.28 (0.77)	Ref	–	–	10.19 (3.21)	Ref	Ref	–
Affected	3.19 (0.98)	− 0.09 (− 0.24–0.06)	–	–	8.59 (4.28)	− 1.6 (− 2.24 to − 0.95)	0.019 (− 0.461 to 0.770)	0.622
Ability to cope (family-same household)								
NA	3.27 (0.85)	0.18 (− 0.46–0.81)	0.003 (− 0.686 to 0.734)	0.946	9.12 (4.18)	2.79 (0.4–5.17)	0.095 (− 0.150 to 6.538)	0.061
Well	3.30 (0.79)	Ref	Ref		10.72 (3.51)	Ref	Ref	–
Poor	3.09 (1.07)	0.21 (0.05–0.36)	− 0.047 (− 0.327 to 0.148)	0.460	6.34 (3.02)	4.39 (3.8–4.97)	− 0.106 (− 1.784 to 0.016)	0.054
Impact (family-different household)								
NA	3.41 (0.79)	0.19 (− 0.06–0.44)	–	–	10.51 (3.33)	0.46 (− 0.62–1.54)	− 0.131 (− 3.966 to 0.733)	0.177
Affected minimally	3.23 (0.82)	Ref	–	–	10.05 (3.38)	Ref	Ref	–
Affected	3.19 (0.96)	− 0.04 (− 0.2–0.12)	–	–	8.45 (4.21)	− 1.6 (− 2.29 to − 0.91)	0.051 (− 0.252 to 1.061)	0.226
Ability to cope (family-different household)								
NA	3.35 (0.81)	0.2 (− 0.04–0.44)	–	–	10.78 (3.35)	4.03 (3.1–4.97)	0.145 (− 0.485 to 3.914)	0.126
Well	3.25 (0.81)	Ref	–	–	10.55 (3.64)	Ref	Ref	–
Poor	3.15 (1.04)	0.1 (− 0.06–0.26)	–	–	6.74 (3.32)	3.81 (3.18–4.43)	− 0.088 (− 1.537 to 0.080)	0.077
Impact (friends)								
NA	3.39 (0.71)	0.06 (− 0.58–0.7)	–	–	8.37 (2.92)	− 2.54 (− 5.22–0.13)	− 0.061 (− 8.204 to 4.099)	0.513
Affected minimally	3.33 (0.70)	Ref	–	–	10.91 (3.20)	Ref	Ref	–
Affected	3.17 (0.98)	− 0.16 (− 0.31–0)	–	–	8.41 (4.03)	− 2.5 (− 3.17 to − 1.84)	0.031 (− 0.438 to 0.950)	0.468
Ability to cope (friends)								
NA	3.40 (0.66)	0.32 (− 0.28–0.92)	0.005 (− 0.617 to 0.694)	0.908	9.11 (3.51)	2.21 (− 0.02–4.43)	0.070 (− 3.335 to 7.763)	0.434
Well	3.34 (0.74)	Ref	Ref		11.17 (3.31)	Ref	Ref	–
Poor	3.08 (1.05)	0.26 (0.11–0.41)	− 0.006 (− 0.219 to 0.195)	0.911	6.90 (3.36)	4.27 (3.71–4.82)	− 0.143 (− 1.904 to − 0.363)	0.004*

*p < 0.05. Model for BRS is adjusted for ethnicity and housing type. Model for HGRS is adjusted for gender, nationality, ethnicity and education institution.

**Table 6:** Association between social life and resilience score.

Variable	Brief Resilience Scale Score	Unadjusted Beta (95% CI)	Adjusted Beta (95% CI)	p-value	Hardy-Gill Resilience Score	Unadjusted Beta (95% CI)	Adjusted Beta (95% CI)	p-value
	Mean ± SD				Mean ± SD			
Impact (social life school)								
NA	3.47 (0.63)	0.05 (− 0.63–0.72)	–	–	9.28 (4.53)	− 1.56 (− 4.41–1.28)	–	–
Affected minimally	3.43 (0.73)	Ref	Ref		10.85 (3.15)	Ref	Ref	–
Affected	3.11 (0.97)	− 0.31 (− 0.46 to − 0.16)	− 0.031 (− 0.258 to 0.142)	0.568	8.34 (4.05)	− 2.5 (− 3.15 to − 1.85)	0.001 (− 0.704 to 0.703)	0.999
Ability to cope (social life school)								
NA	3.47 (0.63)	0.46 (− 0.21–1.12)	0.028 (− 0.460 to 0.924)	0.511	9.28 (4.53)	2.19 (− 0.37–4.75)	− 0.004 (− 2.532 to 2.251)	0.908
Well	3.41 (0.71)	Ref	Ref		11.15 (3.31)	Ref	Ref	–
Poor	3.01 (1.03)	0.4 (0.25–0.55)	− 0.139 (− 0.484 to − 0.020)	0.033	7.09 (3.46)	4.05 (3.49–4.62)	− 0.041 (− 1.107 to 0.466)	0.424
Impact (social life hawker)								
NA	3.27 (0.73)	0.04 (− 0.18–0.26)	–	–	11.28 (4.16)	1.25 (0.35–2.14)	0.165 (− 0.305 to 3.866)	0.094
Affected minimally	3.22 (0.84)	Ref	–	–	10.03 (3.46)	Ref	Ref	–
Affected	3.22 (1.01)	− 0.01 (− 0.17–0.15)	–	–	7.66 (3.73)	− 2.37 (− 3.03 to − 1.71)	0.019 (− 0.608 to 0.917)	0.691
Ability to cope (social life hawker)								
NA	3.25 (0.73)	0.01 (− 0.23–0.24)	–	–	11.18 (4.12)	4.69 (3.79–5.59)	− 0.138 (− 3.559 to 0.574)	0.157
Well	3.21 (0.86)	Ref	–	–	10.11 (3.59)	Ref	Ref	–
Poor	3.24 (1.05)	− 0.02 (− 0.2–0.14)	–	–	6.49 (2.98)	3.62 (2.96–4.28)	− 0.052 (− 1.437 to 0.539)	0.372
Impact (social life family)								
NA	3.28 (0.62)	0.07 (− 0.3–0.45)	–	–	11.48 (4.66)	1.6 (0.01–3.19)	− 0.252 (− 10.655 to 0.913)	0.099
Affected minimally	3.21 (0.87)	Ref	–	–	9.88 (3.65)	Ref	Ref	–
Affected	3.24 (0.94)	0.03 (− 0.12–0.18)	–	–	8.51 (3.96)	− 1.36 (− 2.01 to − 0.71)	0.008 (− 0.564 to 0.688)	0.846
Ability to cope (social life family)								
NA	3.31 (0.62)	0.18 (− 0.19–0.55)	–	–	11.69 (4.69)	5.09 (3.63–6.56)	0.289 (− 0.115 to 11.111)	0.055
Well	3.27 (0.86)	Ref	–	–	10.25 (3.63)	Ref	Ref	–
Poor	3.13 (1.02)	0.14 (− 0.03–0.3)	–	–	6.59 (3.14)	3.65 (3.01–4.3)	0.081 (− 0.251 to 1.644)	0.149

*p < 0.05. Model for BRS is adjusted for ethnicity and housing type. Model for HGRS is adjusted for gender, nationality, ethnicity and education institution.

### Hardy-Gill Resilience Scale Score reflecting the impact of COVID-19 and coping ability

Participants had an average score of 9.23/18 when measured on the HGRS. Mid resilience score was reported by half of the participants (49.3%), followed by low-range resilience (29.0%) and high-range resilience (21.7%) (Table 3).

Over half of the participants (54%) responded that they felt much worse than pre-COVID-19. The question of "Has this event made a permanent change in how you feel about your life?" yielded unchanged with the highest frequency (53%) (Supplementary information Table S1).

The resilience level was not associated with impact on life, social interactions, or social life, even during a pandemic. However, poor ability to cope with school life (adjusted beta = − 0.163, 95% CI − 1.928 to 0.639, p < 0.001), staying home (adjusted beta = − 0.108, 95% CI − 1.611 to − 0.126, p = 0.022), sports (adjusted beta = − 0.116, 95% CI − 1.691 to − 0.197, p = 0.013) and friends (adjusted beta = − 0.143, 95% CI − 1.904 to − 0.363, p = 0.004) were associated with statistically significant low resilience level. The resilience level was not associated with the ability to cope in social life during the pandemic (Tables 4, 5, 6).

## Discussion

The study highlights an important area of concern affecting adolescent students during the COVID-19 pandemic in Singapore. Brief Resilience Scale measured the respondents' general resilience level, while the resilience level measured by Hardy-Gill Resilience Scale was specific to the recent major event such as the COVID-19 pandemic in the present study. Both scales showed a similar proportion of adolescents (approximately 50%) had a normal resilience level. These findings show that the resilience level of the adolescents in this study population was not affected by the COVID-19 pandemic. In addition, the impact of their life settings, social life and social interactions in general and due to COVID-19 had not affected their resilience level. However, participants with lower resilience appeared to cope poorly with school life, sports and friends during the COVID-19 pandemic.

Based on BRS, the resilience level of the participants was higher compared to their Canadian counterparts, with an average score of 3.23 vs. 3.17, as most Canadian students generally had lower resilience scores^26^. The increased resilience level among local participants may be due to Singapore's solid social support system. During the early period of the COVID-19 pandemic, the Singapore government collaborated with community partners and schools to initiate outreach programs and initiatives to support the children and youth. These additional resources enable the teachers to regularly check-in and support the mental well-being and home-based learning of their students^27,28^. Measures are put in to identify high-risk students so that they are closely monitored and receive face-to-face counselling if deemed appropriate or necessary^27^. Youths are also empowered to take charge of their well-being by raising self-awareness of mental health, sharing resources and peer support groups^29,30^.

The findings on resilience level compared with the sociodemographic features of the adolescents denote that the generally lower resilience of adolescents of the Chinese ethnicity and low socioeconomic status is unrelated to the COVID-19 pandemic. The findings are consistent with a local survey in 2011 revealing that Chinese youth were less resilient than their non-Chinese peers. The former also reported a lower level of belonging, fewer friends, less involvement in activities, and less time with family^31^. These are essential predictors of resilience among adolescents, and a lack of these factors may be associated with lower resilience among the Chinese ethnic group.

Participants from junior colleges had higher resilience than their peers in this study. According to the challenge model of resiliency, adolescents exposed to moderate levels of risk learn to overcome ensuing stressors, boosting their resilience^32^. Adolescents in institutes that demand higher performance are regularly exposed to a competitive environment. Such exposure to a stressful environment helps the youth overcome subsequent adverse life experiences, such as during a pandemic. Thus, the disruptions due to the pandemic resulted in less impact on adolescents studying in high-performing institutes such as junior colleges. In addition, a higher resilience is associated with positive academic performance^33^. Those less academically inclined may have difficulty coping with school and study problems with the additional stress of school closure and adapting to remote learning during the pandemic.

Identifying a vulnerable subset of adolescents is essential to design person-centred interventions to strengthen their resilience. Resilience-building programs involve positive adaptation in situations of adversity and focus on strengthening internal (coping skills) or external (family, friends, and community sources) protective factors^34^. Such programs aim to equip adolescents with coping skills and improve their ability to manage daily stressors^35^. School programs that support sustainable education can help to boost confidence and resilience among youth^36^. Sustainable education is aimed to develop individuals who are not only knowledgeable but also empathetic socially responsible and environmentally conscious^36^. Alternatively, technology-based remote support such as chatbots and mobile applications may be used to promote resilience and coping skills in tech-savvy young adults^37,38^. Future research is necessary to assess the effectiveness of these programs and applications on the higher risk adolescents.

While about one in three participants (37%) reported difficulty making it through stressful events, three-quarters of them (76%) reported bouncing back quickly after a setback. The competitive academic environment could be a factor. In 2009, Rebecca et al. reported that Singaporean adolescents faced significantly higher academic stress from self-expectations, other expectations, and overall academic stress than Canadian adolescents^39^. However, the resilience level of participants in general and during the pandemic has not been affected by the impact on their life settings, social life and social interactions. Their resilience could be boosted by the various support schemes such as the buddy system. This support system equips the adolescent students with skills to recover promptly when faced with adversities^40,41^. One such skill that may facilitate bouncing back from hardship is active coping. The active coping technique is associated with positive adjustment and fewer symptoms of mental health problems^42^. Evidence has shown that using active coping strategies such as socializing, engaging in hobbies and exercising during COVID-19 improves psychological distress^15^.

The COVID-19 pandemic had significantly impacted participants’ sports and exercise. The participants reported poor coping with sports, with one in four participants not engaging in physical activities. In a recent systematic review, Bentlage et al. concluded that physical inactivity due to current pandemic restrictions is a significant public health issue, which significantly increases the risks of decreased life expectancy and many physical health problems^43^. A recent study assessing physical activity among adolescents in the USA during the COVID-19 pandemic revealed that higher coping was associated with higher physical activity^44^. Therefore, it is of utmost importance to bolster exercise programs amongst youths. Educational institutes should focus on promoting exercise that can be carried out at home, using digital platforms to cater to the technologically savvy nature of adolescents. Augmented reality software applications convert living spaces into creative exercise landscapes and include a nifty leaderboard and ranking system to promote healthy competition among users. Applications that have gamified the experience of running, introducing community-based challenges and network systems help people encourage their friends and family and promote bonding and social interactions^45^. Evidence shows active gaming improves physical activity in adolescents^46^. Encouraging such apps can help youths find the motivation to exercise, especially during the trying times of this pandemic. With the end of the pandemic, adolescents can be urged to take up exercises that promote environmental friendliness, such as biking or walking. Such physical activity promotes physical and mental well-being among adolescents^47^.

The COVID-19 has affected social interactions^48^. This is consistent with the participants having poor coping with school and friends and reporting a significant impact on school life and socializing with friends during the pandemic. Less resilient participants reported poor coping. This trend can likely be attributed to the long circuit breaker period, mandatory lockdowns and distancing measures, where adolescent students had to stay home^49^. These measures restrict the adolescents' daily and leisure activities, such as sports or hobbies or even hanging out with peers and friends. The school closures have resulted in many adolescents not attending school and leaving them to transition to virtual and distance learning^50,51^. These measures are particularly difficult for adolescents, who at this developmental stage rely heavily on their peer connections for emotional support and social development, which are essential components of coping and resilience^52^. While virtual solutions to this problem exist, such as using social networking apps like Discord, Instagram or WhatsApp, the depth and level of interaction fail compared to traditional physical interactions.

As the COVID-19 pandemic has resulted in more staying at home, this creates a greater opportunity for the family to spend more time with their families. However, there have been reports of increased negative feelings at home, as stress and anger can be pent up without release^53^. Frustration and anger can become more common, and thus family tension can arise, resulting in difficulty for adolescents to cope at home. Participants’ social interaction with the family was affected significantly by the COVID-19 pandemic. The former reported poor coping to the homestay, and 35% of the participants found it challenging to cope with living with family members. A local study among young adults showed that family functioning was significantly associated with intergenerational communication and satisfaction with social support in a pandemic. The study informed that young adults with balanced levels of cohesion and flexibility in their families were more likely to cope with the pandemic's psychological impacts^54^. Locally, early childhood programs have been initiated to equip parents with knowledge and skills to nurture their children's early development and foster a stronger family bonding during the pandemic^55^. Future research is needed to assess if such programs can boost resilience and coping among young adults.

This is the first study assessing resilience in adolescent students in a multi-ethnic, multi-cultural urban society during the COVID-19 pandemic. Using both Brief Resilience Scale and Hardy-Gill Resilience Scale, the variation of resilience level of the participants in general and specific to COVID-19 was assessed. As the current pandemic continues, the findings of this study add to the emerging literature on the impact of the COVID-19 pandemic on adolescents and their resilience.

The current study has several limitations. Given that the resilience level was measured during the COVID-19 pandemic, a change in the resilience level of the target population compared with the pre-and post-pandemic could not be assessed. An extension of this study would measure the change in resilience levels of a group of selected participants across time. Measuring this change over time would also give a better insight into the effectiveness of existing solutions that aim to help students cope with the effects of the pandemic. The study did not compare the change of social life and coping behaviour of the adolescents due to COVID-19, as data on these aspects prior to the pandemic was unavailable. Questions about this information were not included in the questionnaire to avoid making it lengthy and keep the participants focused in completing the survey. Nevertheless, the study captures a snapshot of life experiences of the adolescents amidst the pandemic in Singapore in 2021.

Another limitation was that the validity and reliability of the bespoke questionnaire and the resilience scales were not determined in the local population. However, the resilience scales employed have been used in previous studies and validated in similar settings^22,23,25^. In addition, the data collection method relied on self-reporting from adolescents. Hence the data collected is susceptible to subjectivity, resulting in response bias. Ideally, a mixed method comprising qualitative research and complemented by scores from validated scales, which allow triangulation of the results, will be most suited to seek answers to the research questions. The complexities of human psyche, behaviour and the context of the responses of the adolescents to demonstrate their coping behaviour and resilience levels is better captured via interviews or other modalities using qualitative research methodology. Nevertheless, the circumstances amidst the pandemic which restricted person-to-person interactions did not allow the implementation of such a research method in the institution. Hence, the combined results from the scales would be a pragmatic approach to reflect on the impact of the pandemic on the social behaviour and resilience of the adolescents.

Convenience sampling via networking and snowballing was used in recruiting the participants. Although this approach facilitated a timely recruitment and data collection, which was necessary for a rapidly evolving pandemic, selection bias may limit generalisability to the general Singapore adolescent population. The response rate of the study could not be determined as the survey was delivered online and number of target subjects who accessed the web portal was not tracked. There is a potential for selection bias from non-responses, but the extend that this bias existed could not be assessed due to lack of data on non-response rate. The study sample consisted mainly of Chinese ethnicity, and views of the non-Chinese may have been less represented. However, the distribution of the ethnicity in the study population is representative of the Singapore population.

## Conclusion

Approximately half of the adolescent students demonstrated resilience during the COVID-19 pandemic. However, one-third of them had low resilience based on the scale scores. Those of Chinese ethnicity and low socioeconomic status were less resilient than others. Yet, this was unrelated to COVID-19 pandemic. The social and physical activities of most adolescents were minimally affected by the pandemic. However, adolescents with lower resilience tended to have lower coping due to disruptions during the COVID-19 outbreak. The study did not compare changes in the social life and coping behaviour of the adolescents due to COVID-19, as data on these aspects prior to the pandemic was unavailable. The extent of the coverage of the target participants is also limited by the nature of the online anonymous survey. The results highlight the need to identify at-risk adolescents who are not coping well with their daily activities. Special attention can be directed to those with specific sociodemographic risk factors. Healthcare providers, schools, and social service agencies should collaborate to identify at-risk adolescents and design interventions to boost their resilience and coping skills.

## Supplementary Information

Supplementary Information.

## Data Availability

The data that support the findings of this study are available from the corresponding author upon reasonable request. The datasets analysed during the study are available from the corresponding author on request.

## Abbreviations

COVID-19: Coronavirus disease 2019
BRS: Brief Resilience Scale
HGRS: Hardy-Gill Resilience Scale
CI: Confidence Interval
SD: Standard deviation
SE: Standard Error
HDB: (Singapore) Housing Development Board
