# Supplementary material for: Resilience and coping behaviour among adolescents in a high-income city-state during the COVID-19 pandemic
Source: Sci Rep. 2023 Mar 11;13:4061. doi: 10.1038/s41598-023-31147-0 (PMC10006555; doi:10.1038/s41598-023-31147-0)
Supplement: Supplementary file 1 — Supplementary Information. [file 41598_2023_31147_MOESM1_ESM.pdf]

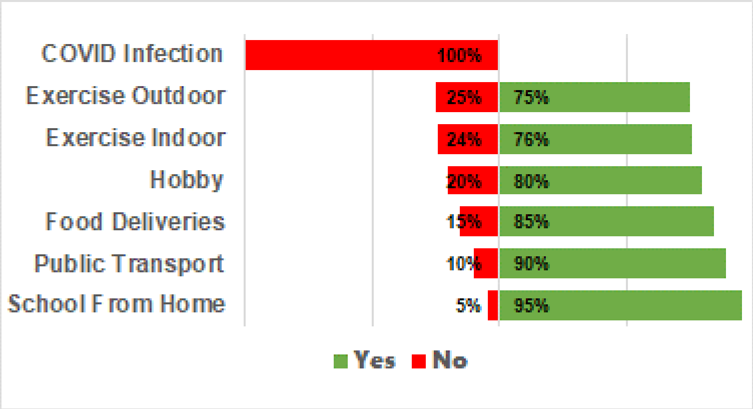

Figure S1: Impact of COVID-19 on daily activities

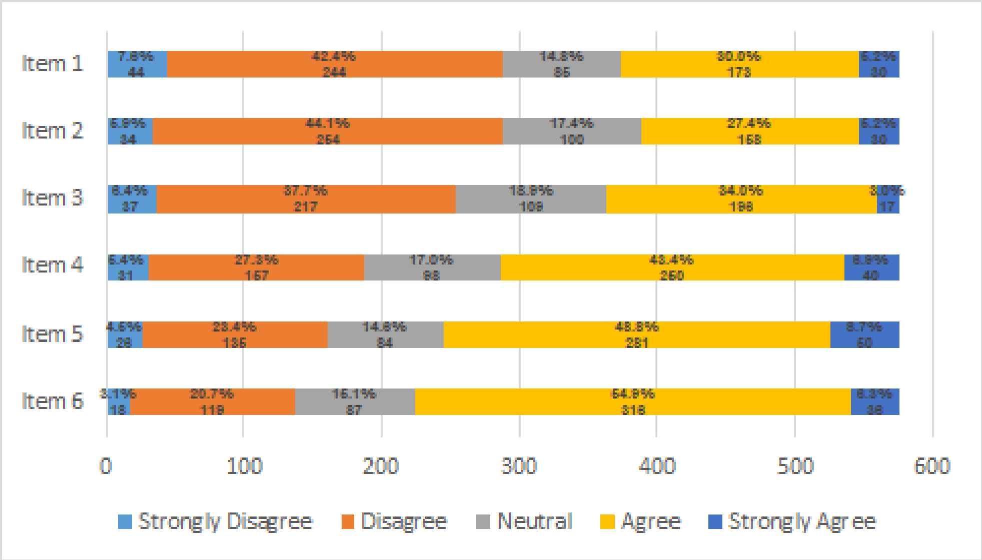

Item 1-3: Negatively Focused

Item 1: I tend to take a long time to get over set-backs in my life

Item 2: It is hard for me to snap back when something bad happen

Item 3: I have a hard time making it through stressful events

Item 4-6: Positively Focused

Item 4: I usually come through difficult times with little trouble

Item 5: It does not take me long to recover from a stressful event

Item 6: I tend to bounce back quickly after hard times

Figure S2: Distribution of Student's Response for Items on Brief Resilience Scale

Table S1a: Distribution of Student’s Response for Items on Hardy Gill Resilience Scale (Item 1-3)

Item	Great Deal	Quite a bit	A Little	Not Affected
Item 1	69 (12.0%)	198 (34.4%)	233 (40.4%)	76 (13.2%)
Item 2	82 (14.2%)	202 (35.1%)	231 (40.1%)	61 (10.6%)
Item 3	73 (12.7%)	236 (41.0%)	223 (38.7%)	44 (7.6%)

Table S1b: Distribution of Student’s Response for Items on Hardy Gill Resilience Scale (Item 4)

Item	Few days	Few weeks	Few months	A year or more	Not better yet
Item 4	90 (15.6%)	119 (20.7%)	206 (35.8%)	131 (22.7%)	30 (5.2%)

Table S1c: Distribution of Student’s Response for Items on Hardy Gill Resilience Scale (Item 5)

Item	Less than a month	1 to 6 months	6 months to 2 years	More than 2 years
Item 5	39 (6.8%)	82 (14.2%)	451 (78.3%)	4 (0.7%)

Table S1d: Distribution of Student’s Response for Items on Hardy Gill Resilience Scale (Item 6-9)

Item	Yes		No
Item 6	460 (79.9%)		116 (20.1%)
Item 7	499 (86.6%)		77 (13.4%)
Item 8	270 (46.9%)		306 (53.1%)
	Better	Worse	
Item 9	124 (45.9%)	146 (54.1%)	

Item 1: After this event, how much worse did you feel than before it happened?

Item 2: After this event, how much more discouraged were you?

Item 3: After this event, how much harder was it to get everyday things done?

Item 4: After this event, how long did it take until you started to feel better again?

Item 5: How long ago did this event occur?

Item 6: As a result of this event, have you stopped doing some activities that were important to you?

Item 7: As a result of this event, have you started doing some activities that have become important to you?

Item 8: Has this event made a permanent change in how you feel about your life?

Item 9: (Only if Yes to 8.). Is that change for the better or for the worse?
